# Ensemble of EfficientNets for the Diagnosis of Tuberculosis

**DOI:** 10.1155/2021/9790894

**Published:** 2021-12-14

**Authors:** Mustapha Oloko-Oba, Serestina Viriri

**Affiliations:** School of Mathematics, Statistics and Computer Science, University of KwaZulu-Natal, Durban 4000, South Africa

## Abstract

Tuberculosis (TB) remains a life-threatening disease and is one of the leading causes of mortality in developing regions due to poverty and inadequate medical resources. Tuberculosis is medicable, but it necessitates early diagnosis through reliable screening techniques. Chest X-ray is a recommended screening procedure for identifying pulmonary abnormalities. Still, this recommendation is not enough without experienced radiologists to interpret the screening results, which forms part of the problems in rural communities. Consequently, various computer-aided diagnostic systems have been developed for the automatic detection of tuberculosis. However, their sensitivity and accuracy are still significant challenges that require constant improvement due to the severity of the disease. Hence, this study explores the application of a leading state-of-the-art convolutional neural network (EfficientNets) model for the classification of tuberculosis. Precisely, five variants of EfficientNets were fine-tuned and implemented on two prominent and publicly available chest X-ray datasets (Montgomery and Shenzhen). The experiments performed show that EfficientNet-B4 achieved the best accuracy of 92.33% and 94.35% on both datasets. These results were then improved through Ensemble learning and reached 97.44%. The performance recorded in this study portrays the efficiency of fine-tuning EfficientNets on medical imaging classification through Ensemble.

## 1. Introduction

According to the World Health Organization (WHO), tuberculosis (TB) is one of the leading causes of death [1, 2]. It is most prevalent in developing countries due to the high rate of economic distress [3]. TB can be treated if detected early to avert its further spread and mortality [4]. Early diagnosis of TB requires a reliable screening procedure and accurate interpretation of the screening outcome. To this end, chest X-ray (CXR) has been recommended by the WHO for screening pulmonary (lungs) abnormalities due to its high sensitivity, wide availability, and relatively less expensive. Despite this recommendation, adequate medical resources and skilled radiologists required to efficiently screen patients and interpret results are limited in high TB burden countries [5, 6]. Several algorithms varying from hand-crafted to support vector machines (SVMs) and convolutional neural networks (CNNs) have been deployed to automate TB screening in tackling these limitations. However, our focus is on the CNN algorithms.

Amongst these algorithms, CNNs have demonstrated promising performance, as evident in the literature primarily in transferring models trained on large datasets (ImageNet [7] and CIFAR-10 [8]), to medical imaging with fewer datasets. One of the challenges in the medical field is the lack of reliable annotated datasets and class imbalance; hence, transferring the weights of deep models helps address these challenges since CNN algorithms often depend on large datasets to learn various discriminating features contributing to attaining good results [9].

To date, many CNN models have been developed to address the challenges of TB detection, some of which are discussed here, ranging from the ones trained from scratch to pretrained CNNs and Ensemble of them.

The CNN architecture proposed in [10] is trained from scratch to diagnose TB. The architecture comprises five convolutional layers, each preceded by the pooling layers, and then the final output layer to obtain 86.2% accuracy and AUC of 0.925%. The authors incorporate Grad-CAMS and Saliency map visualizers to validate the output of the model classification and identify areas where TB was visible on the CXR. The importance of a saliency map is further emphasized as a valuable tool to review and interpret the model decision.

A technique based on deep CNN models is presented in [11] for screening CXR to detect TB. Artificial CXR images were generated using deep convolutional generative adversarial networks (DC-GANs). These images and the CXR images from popular datasets were segmented into only the lung fields using UNET. The segmented CXR images are then used to train Vgg16 and InceptionV3 and then Ensemble both for classification. The Ensemble model achieved 97.1%, while the individual model obtained 93.8% and 96.3%, respectively. The authors affirm that the performance of the proposed model is impacted by the preprocessing technique, segmentation, augmentation, image denoising, and hyperparameter optimization.

In [12], VGG16, a variant of VGGNet [13], was employed as a feature extractor to extract discriminating features from CXR, and a logistic regression classifier was trained to predict the normal and infected images. Preprocessing techniques such as augmentation and contrast limited adaptive histogram equalization were applied to boost the quality of the CXR. The authors conclude that a large database of labeled CXR would assist in building a deeper and robust computer-aided detection system from scratch. The study shows the effectiveness of transferring the weights of a pretrained model to the medical imaging domain where there are limited annotated datasets.

GoogLeNet [14] was fine-tuned in [15] to classify different manifestations of TB as consolidation, pulmonary edema, cardiomegaly, pneumothorax, or pleural effusion. Image preprocessing was applied to normalize the CXR with shorter dimensions, while the CXR with larger dimensions was padded before training the model. The performance of the model was evaluated to determine its AUC score, sensitivity, and specificity. The model obtained 0.964 AUC and 91% for both specificity and sensitivity, which are further compared with the result of 2 board-certified radiologists.

The study presented in [16] used the pretrained CNN to confirm the presence of TB on CXR. In the study, the authors conducted two experiments by applying preprocessing such as data augmentation to CXR in the first experiment, while the second experiment was performed directly on the CXR. The model achieved an accuracy of 81.25% in the first instance and 80% in the second instance. This performance is low compared to what could be acceptable due to the sensitive nature of TB. However, it has shown data augmentation to increase data samples where necessary and proves that the more data samples you have, the more features can be learned and better the performance.

Ensemble classifier is presented in [17] for detecting TB abnormalities in the CXR. Firstly, three pretrained CNN models are trained individually with different hyperparameters to classify the CXR into normal and abnormal classes. The three models are then combined through Ensemble to improve the classification. The results are then compared, and the Ensemble model performed better than each model, achieving 86.42%.

Pretrained CNN models were customized and proposed in [18] to classify CXR abnormalities. The study applied data augmentation to generate more CXR. After the image preprocessing, CapsNet [19], VGG16, and AlexNet [20] were trained to classify the CXR into normal and abnormal classes. The classifiers were evaluated using different metrics, and the CapsNet performed best above the others concerning affine transformations. Lopes and Valiati [21] proposed three approaches (Bag of features, Simple feature extractor, and Ensemble) as feature extractors and compared their performance to detect TB.

The Ensemble in the study is made up of VggNet, ResNet [22], and GoogLeNet. The CXR was first downsampled to suit the different approaches excluding Bag of features; even though downsampling could impact the performance due to vital detail loss, experiments were then performed for all the proposed methods, and the Ensemble outperformed the other two approaches, achieving 84.6%. This performance could have been impacted due to downsampling, class imbalance, and limited datasets.

The study in [23] proposed an Ensemble of pretrained CNN models toward learning modality-specific features from different CXR collections. The knowledge learned through modality specific is transferred and fine-tuned for TB detection tasks on the Shenzhen CXR dataset. The predictions of the best performing models are combined using different ensemble methods to demonstrate improved performance over any individual model in classifying the CXR as infected or healthy. The evaluation of the proposed model achieved 94.1% accuracy.

Another study that makes use of Ensemble models is proposed in [24]. This study extracted distinctive features from edge detected images and raw CXR images for TB detection. Image preprocessing was applied before developing different classifiers to represent Ensemble and applied to both image categories. The performance was evaluated, and the Ensemble model performed best at 89.77% accuracy with 90.91% sensitivity.

The study presented in [25] is a proposed method that fine-tuned five pretrained CNNs as feature extractors to diagnose TB automatically. The CXR was collected with the patient's demographic details such as weight, height, weight, age, and gender. The authors conducted two experiments that compare the performance of the CXR with demographic information and the CXR without demographic details. The first experiment shows higher performance in terms of AUC and sensitivity over the second experience. The result proves that incorporating demographic information can positively impact performance.

In [26], VggNet and AlexNet architectures were modified to screen TB on CXR and classify them into healthy and unhealthy classes. The architectures were independently trained on the Montgomery and Shenzhen datasets to evaluate the performance of each model. VggNet achieved 81.6% classification accuracy, a slight superiority over the AlexNet architecture at 80.4%.

Deep CNN models proposed in [27] were integrated with the handcraft technique through Ensemble learning as feature extractors from CXR. The features extracted were then used as inputs to train a classifier for detecting infected CXRs. The model was evaluated to compare the performance of both methods, and the Ensemble model performed better at 0.99 AUC.

In [28], an automatic deep learning algorithm for the diagnosis of active lung TB was presented to streamline the process of screening and detection. The algorithm was developed using the CNN comprising about 27 layers. The CXR images were resized; then, a geometric and photometric operation was applied to them before passing them on to the CNN architecture for training. The algorithm was evaluated on two public datasets and four custom datasets compared with certified radiologists and physicians' interpretations. The algorithm performance shows significant performance compared to the radiologist and physician's performance on all the datasets.

A hybrid model (MoblieNet-AEO) was proposed in [29] to classify CXR images as TB and Not-TB. The proposed model first employed MobileNet as feature extraction and then used the Artificial Ecosystem-Based Optimization (AEO) as the feature selector. The model was trained with the Shenzhen dataset and a private dataset to achieve 90.2% and 94.1% accuracy on both datasets.

Although several computer-aided detection models have been developed to diagnose TB, low accuracy and sensitivity are still major concerns that lead to misdiagnosis. Existing models are also computationally complex and expensive, which make them unaffordable in a low-budget economy. All these serve as motivation to propose an efficient system suitable and accessible for low-income economies. The contributions of this work are summarized as follows:

1. Implementation of state-of-the-art EfficientNets to develop an effective and inexpensive TB detection system. It is the first time the EfficientNet model is being ensembled to classify CXR images for tuberculosis diagnosis.
2. The proposed model achieved high sensitivity and accuracy.
3. The model improved accuracy through Ensemble learning (Bagging).
4. Finally, two benchmark datasets (Shenzhen and Montgomery) were used to evaluate the performance of our model. Despite the challenges of limited annotated datasets and class imbalance in medical fields, our approach produces significant improvements in TB classification accuracy over the related methods.

The rest of the work is structured as follows: Section 2 presents detailed data and methodology explored in this study. The experimental results and discussion are provided in Section 3, while Section 4 concludes and gives insight into future direction.

## 2. Data and Methods

### 2.1. Dataset

In this study, all the experiments were performed with the two public CXR datasets provided by the Department of Health and Human Service, Montgomery Country, Maryland, USA, and Shenzhen No. 3 People's Hospital in China [30]. These datasets were deidentified and exempted from IRB review. Both datasets described below can be accessed from the website https://lhncbc.nlm.nih.gov/publication/pub9931.

**Figure 1:**
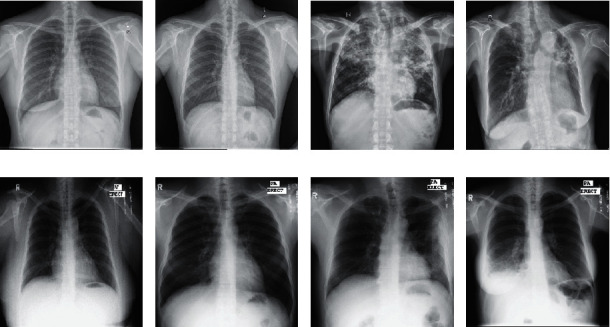
Training and validation CXR from both Shenzhen and Montgomery datasets. (a) Normal CXR of a 38 yr female. (b) Normal CXR of a 22 yr male. (c) Infected CXR of a 56 yr male. (d) Infected CXR of a 78 yr female. (e) Normal CXR of a 40 yr female. (f) Normal CXR of a 33 yr male. (g) Infected CXR of a 47 yr male. (h) Infected CXR of a 49 yr female. (a–d) Samples from the Shenzhen dataset. (e–h) Samples from the Montgomery dataset [30].

1. The Montgomery County dataset: this dataset is a TB-specific frontal CXR dataset provided by the National Library of Medicine in conjunction with the Department of Health Services, Maryland, USA, for research intent. This dataset is composed of 58 abnormal samples and 80 normal samples. The naming of each sample ends with either 0 denoting normal sample or 1 denoting abnormal sample. All samples are 4020 × 4892 pixels and are available in portable network graphic (png) file format. This dataset is accompanied by clinical readings that detail each sample regarding age, sex, and manifestations.
2. The Shenzhen dataset: this dataset is a TB-specific frontal CXR provided by Shenzhen No. 3 Hospital in Shenzhen, Guangdong, China, and is publicly available for research. The dataset is made up of 336 abnormal samples and 326 normal samples. The naming of each sample ends with either 0 denoting normal sample or 1 denoting abnormal sample. All samples are approximately 3000 × 3000 pixels saved in portable network graphic (png) file format. This dataset is accompanied by clinical readings that detail each sample concerning sex, age, and diagnosis. Some of the CXR in both datasets are shown in Figure 1.

### 2.2. Preprocessing

Image preprocessing is crucial in a deep-learning task. Since images are provided in different sizes, the input images need to be resized to conform to the different CNN models while preserving the important features. Also, the CNN model generally requires a large dataset to properly learn discriminating features suitable for making predictions and obtaining a reasonable performance [9]. Medical imaging is usually limited; hence, data augmentation [31] is applied in this study to generate additional unique CXR images. The data augmentation approach contributes toward controlling overfitting. Overfitting is a phenomenon where the model performs well on the training set but poorly on the new (unseen) test set. Contrast limited adaptive histogram equalization (CLAHE) [32] is also applied on the CXR to improve the visibility quality of the images. The augmentation types applied to the CXR images are presented in Table 1, while Table 2 shows the parameter values used to perform the CLAHE operation.

### 2.3. Transfer Learning

**Figure 2:**
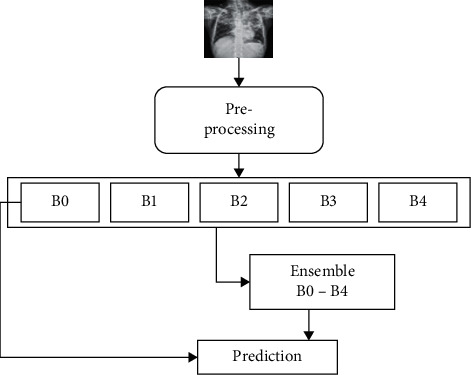
Block structure of the proposed model.

In this study, we adopt the concept of transfer learning. This phenomenon leverages the pretrained CNN model on large datasets (millions) to learn features of the target (diagnosing TB from the CXR in this case) with a limited dataset. In other words, we transfer the rich discriminative features learned by the deep CNN models on the ImageNet [7] dataset to our CXR dataset. The number of deep CNN model parameters increases as the network gets deeper to achieve improved efficiency. Hence, it requires many datasets for training, thereby making it computationally complex. So, applying these models directly on small and new datasets results in feature extraction bias, overfitting, and poor generalization. We, therefore, modified the pretrained CNN and fine-tuned its structure to suit the CXR dataset. The concept of transfer learning is computationally inexpensive, achieves less training time, overcomes limitations of the dataset as in the medical domain, improves performance, and is faster than training a model from scratch [33]. The pretrained CNN model fine-tuned in this work is the EfficientNets [34], and the structure of the proposed method is represented in Figure 2.

### 2.4. EfficientNet Architecture

**Figure 3:**
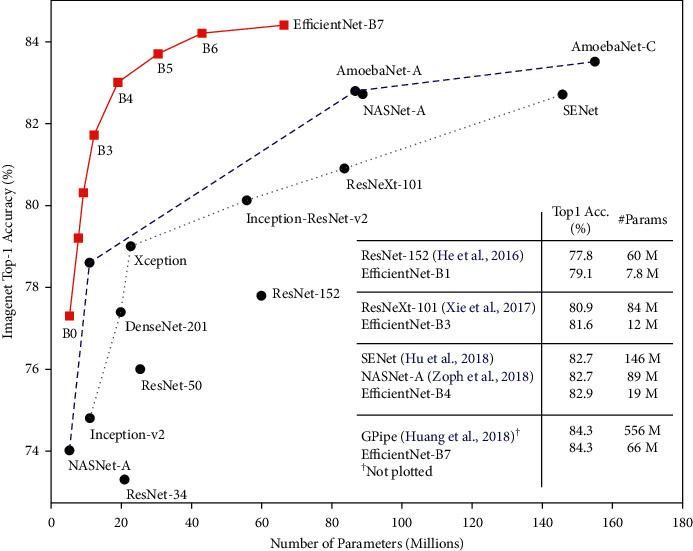
Comparison of EfficientNet model size with other models on ImageNet [34].

**Figure 4:**
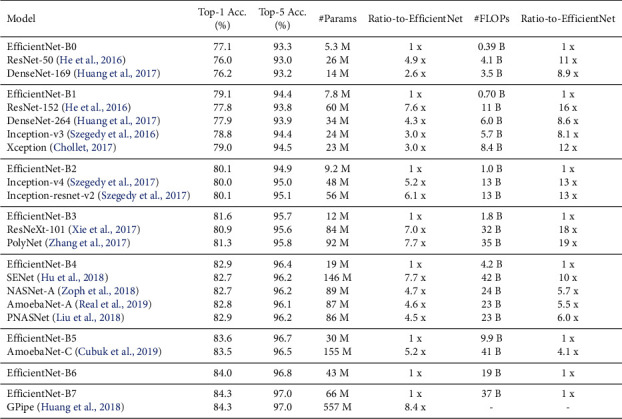
Performance of EfficientNets vs state-of-the-art models on ImageNet [34].

EfficientNet [34] is a lightweight model based on the AutoML framework [35] to develop a baseline EfficientNet-B0 network and uniformly scaled up the depth, width, and resolutions using a simplified and effective compound coefficient to improve EfficientNet models B1–B7. The models performed efficiently and attained superiority over the existing CNN models on the ImageNet datasets, as shown in Figures 3 and 4, respectively. EfficientNets are smaller with few parameters, faster, and generalize well to obtain higher accuracy on other datasets popular for the transfer learning task. The proposed study fine-tuned EfficientNet models B0–B4 on CXR to detect TB. Due to limited annotated datasets in medical imaging, the data augmentation technique is applied to generate additional unique CXR images to control overfitting. In transferring the pretrained EfficientNets to the CXR dataset, we fine-tuned the models by adding a global average pooling (GAP) to reduce the number of parameters and handle overfitting. Two dense layers follow the GAP with a ReLU activation function and a dropout rate of 0.4 before a final dense layer that serves as output with SoftMax activation function to determine the probabilities of the input CXR representing the normal and infected classes. The SoftMax activation function is given as

(1) $$\begin{array}{c} \sigma {\left(q\right)}_{i}=\frac{e^{q_{i}}}{\sum_{y=1}^{N}e^{q_{y}}}. \end{array}$$

where *σ* is the SoftMax activation function, *q* represents the input vector to the output layer *i* depicted from the exponential element *e*^*q*_*i*_^, *N* is the number of classes, and *e*^*q*_*y*_^ represents the output vector of the exponential function.

It is understood that too many iterations could lead to model overfitting, while too few iterations can cause model underfitting; hence, we invoked an early stopping strategy. About 120 training iterations were configured and then set up the early stopping to terminate the training once the performance stops improving on the hold-out validation set. The early stopping, L2 regularization, data augmentation, and dropout were implemented to control overfitting. The EfficientNet B0–B4 models were trained for 40 iterations (epochs) using a stochastic gradient descent (SGD) optimizer with a 0.0001 learning rate. The batch size for each iteration was 22, and momentum equals 0.9 and L2 regularizer. All these configurations help to control overfitting. At the same time, categorical cross-entropy is the loss function used to update weights at each iteration. Hyperparameters used were carefully evaluated and found to perform optimally. The optimizer employed is defined as

(2) $$\begin{array}{c} \alpha =\;\alpha -\;n\cdot \Delta_{\alpha \;}J\left(\alpha ;x^{\left(i\right)};y^{\left(i\right)}\right), \end{array}$$

where Δ_*α* _*J* is the gradient of the loss w.r.t *α*, *n* is the defined learning rate, *α* is the weight vector, while *x* and *y* are the respective training sample and label, respectively.

### 2.5. Ensemble Modeling

Ensemble learning is the terminology for techniques that incorporate multiple models for decision making. The ultimate goal of Ensemble is such that by combining multiple models, the errors of a single model can be corrected (compensated) by other models, thereby making the overall result (prediction and classification) of the Ensemble better than any single participating model [36]. The concept of Ensemble is mainly considered the interpretation of machine learning for the intelligence of the majority.

In recent times, Ensembles are regarded as state-of-the-art methods for solving a more significant number of machines and deep learning challenges [37]. In other words, weighing and aggregating many individual viewpoints will be better than deciding on the judgment of a single individual.

(1) Strength of Ensemble learning: the efficiency of Ensemble models for obtaining improved performance could be due to the following [36, 38]:
  Avoidance of overfitting: algorithms often find ways to perfectly predict the training set in a few datasets even though poor prediction is made on the test set. So when a different hypothesis is averaged, the risk of selecting the wrong hypothesis is minimized.
  Computational expediency: individual models can get stuck in a local minimum, but through (Ensemble) combining models, the risk of local minimum is decreased as other models can get past a certain local minimum.
  Representation: Ensemble models help improve the scenario where the optimal hypothesis is outside the scope of any individual model.
  Class imbalance and dimensionality: they are major problems that affect the equality of results and are well handled by Ensemble model through resampling.
(2) Building an Ensemble model: building an Ensemble model culminates in methodology selection to train the individual participating models and select a suitable process for combining the individual predictions (output). The high diversity and predictive performance of a model are essential in selecting participating models [38]. Other considerations for model selections include input and output manipulations, partitioning, and hybridization. Furthermore, the methods of integrating all individual outputs into a single final result are crucial. There are different Ensemble types based on the generation process of different models and a combination strategy. The most prominent Ensemble types are Bagging [39], Boosting [40], and Stacking [41]. In this study, the Bagging method of Ensemble is implemented. This method is also known as Bootstrap Aggregation.
(3) Bagging [39] is an effective method for creating Ensembles of independent models. Many individual models are trained independently with a sample of data derived from the original datasets as replacements. By independently, we mean the training and output of a particular model do not affect the others. To ensure diversity, each replica sample has the same number of samples as the original dataset. Thus, some data samples may appear for the replica, while others may not or appear more than once for training individual models. Once the classification output of the models is obtained, bagging then combines them through majority voting to obtain the final prediction.  Algorithm 1 presents bagging pseudocode. The bagging method is employed in this study because of the following:
  It is suitable for a task with small training datasets.
  It can achieve low variance without impacting the bias.
  It handles classification tasks well
  It has the ability to obtain improved accuracy.

## 3. Results and Discussion

Various EfficientNet (B0–B4) variants were fine-tuned on both Shenzhen and Montgomery TB-specific CXR datasets to detect TB. Each dataset is split into a 75% training set and 25% test set, respectively. The experiments were entirely performed using the Keras deep learning framework running on the TensorFlow backend. Each model was trained in the cloud on the Tesla graphics processing unit (GPU) and made available through the Google Collaboratory framework by Google. The Collaboratory framework provides up to 12 GB random access memory (RAM) and about 360 GB GPU in the cloud for research purposes. The models were evaluated using the popular evaluation metrics (accuracy, sensitivity, specificity, and area under the curve). The way these evaluation metrics are determined is given as follows:

(3) $$\begin{array}{c} \text{accuracy}=\;\frac{\mathrm{TP}\;+\text{ TN}}{\mathrm{TP}\;+\;\mathrm{FP}\;+\;\mathrm{TN}\;+\text{ FN}},\\ \text{sensitivity}=\;\frac{\mathrm{TP}}{\mathrm{TP}\;+\text{ FN}},\\ \text{specificity}=\;\frac{\text{TN}}{\mathrm{TN}\;+\;\mathrm{FP}},\\ \text{TPR}=\text{ sensitivity}=\;\frac{\mathrm{TP}}{\mathrm{TP}\;+\text{ FN}},\\ \text{FPR}=1-\text{ specificity}=\;\frac{\mathrm{FP}}{\mathrm{FP}\;+\text{ TN}}, \end{array}$$

where TP = true positive, FP = false positive, TN = true negative, FN = false negative, TPR = true-positive rate, and FPR = false-positive rate.

**Figure 5:**
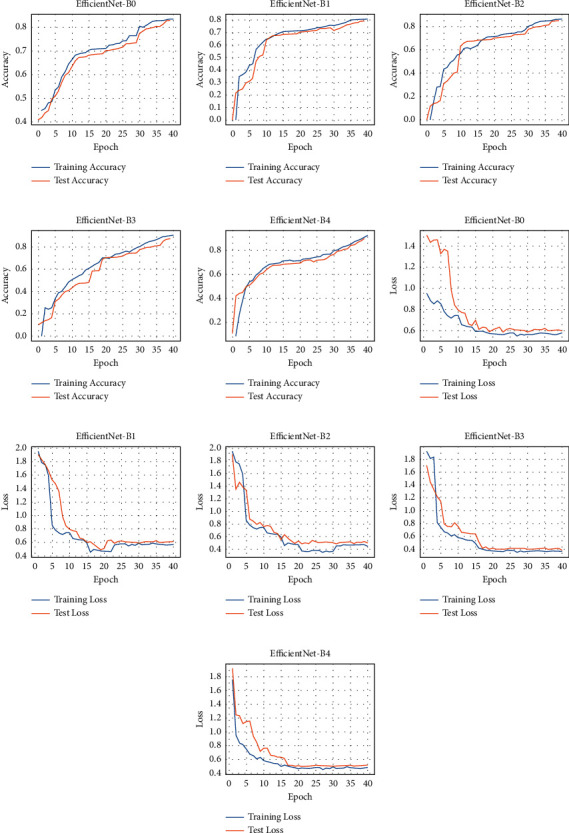
Accuracy and loss for the Shenzhen dataset: (a–e) performance accuracy of each EfficientNet and (f–j) the corresponding loss.

**Figure 6:**
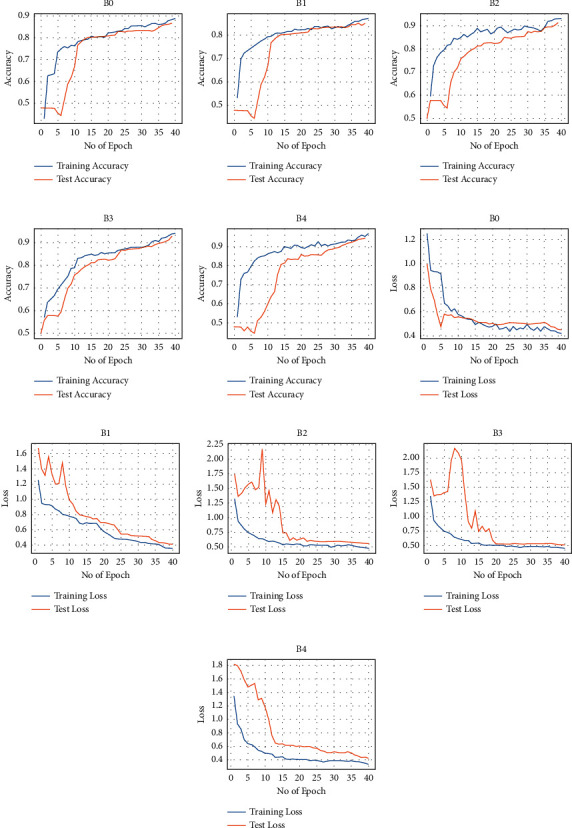
Accuracy and loss for the Montgomery dataset: (a–e) performance accuracy of each EfficientNet and (f–j) the corresponding loss.

The experimental results showing each dataset's accuracy and loss are presented in Figures 5 and 6. The various EfficientNet models converge at the 40th iteration (epoch); then, the accuracy is determined using the test set. The models performed well at extracting and learning discriminative features from the CXR. Precisely, EfficientNetB4 attains the best accuracy (92.33% and 94.35%) for both datasets over the other variants. The performance details of the experiments are illustrated in Table 3, depicting accuracy, sensitivity, specificity, and AUC.

**Figure 7:**
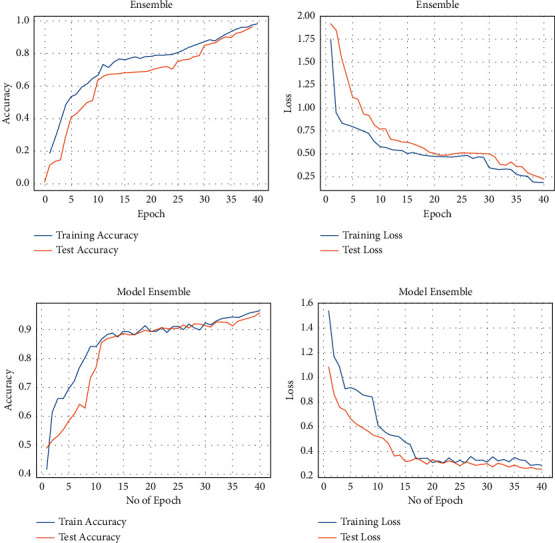
Accuracy and loss of the Ensemble model: (a, b) performance on the Shenzhen dataset and (c, d) performance on the Montgomery dataset.

Despite achieving good performance, we further build an Ensemble model comprising the best performing individual models (B2, B3, and B4) from the initial experiments to improve accuracy. The performance of the three EfficientNets was averaged to build an Ensemble to classify the CXR. The Ensemble model outperformed the individual variants of EfficientNets in diagnosing TB, achieving 95.82% for the Montgomery dataset and 97.44% for the Shenzhen dataset. The accuracy and loss plots for the Ensemble model are presented in Figure 7. Also, the model obtains the highest sensitivity for TB detection.

One of the main advantages of EfficientNets is that they are smaller with fewer parameters and faster and generalize well on transfer learning datasets [34]. EfficientNet-B0 had the least performance, as shown in Table 3, despite having the fewest parameters. This low performance could result from image downsampling to conform to the model's 224 × 224 input size. A detailed number of parameters and input size for each EfficientNet are provided in Table 4. We observed that performance improves as the model gets deeper. For the Montgomery dataset, EfficientNet-B0 started poorly. It only began to converge from the 11th iteration, although with little noise, until the 25th iteration, where it began to stabilize until the 40th iteration. Overfitting starts to occur after the 40th iteration; hence, early stoppage was invoked. On the contrary, the training progressed well for the Shenzhen dataset but only began to overfit after the 40th iteration. Generally, the models performed better on the Shenzhen dataset. This attribute could be due to the increased dataset and balanced class ratio of normal to abnormal CXR. One of the challenges faced during the experiments was determining the best combinations of hyperparameters suitable for these models and overfitting due to limited data samples. Hence, we explored data augmentation and other regularization techniques (dropout) to combat overfitting as much as possible. The result of the proposed method compared with the related Ensemble study is presented in Table 5.

## 4. Conclusion

**Figure 8:**
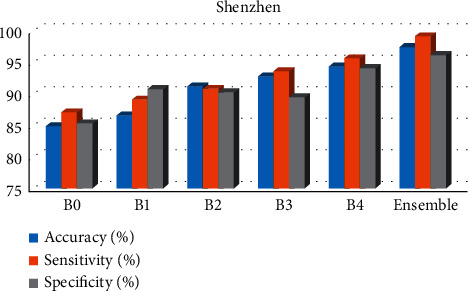
Best results achieved on the Shenzhen dataset.

This study investigated and implemented EfficientNet models for automatic diagnosis of TB on the two most prominent and publicly available CXR datasets. EfficientNets that achieved state-of-the-art performance over other architectures to maximize accuracy and efficiency were explored and fine-tuned on CXR images. The fine-tuning technique is a valuable way to take advantage of rich generic features learned from large dataset sources such as ImageNet to compliment the lack of annotated datasets affecting medical domains. The experimental results show the effectiveness of the EfficientNets in extracting and learning distinctive features from the CXR images and then classifying them into a healthy or infected class. Out of the five EfficientNet variants explored in this study, the EfficientNet-B4 outperformed among the others, as evident in Table 3 and depicted in Figure 8. The achieved result is improved through Ensemble of the best three (B2, B3, and B4). It is worthy of note that exploring EfficientNets for the classification of TB images helps save time while preserving accuracy. However, one of the major limitations of the proposed approach is training the model on small datasets and training on images with low resolutions. These limitations can easily result in significant overfitting. Hence, it is essential to explore effective image preprocessing techniques to overcome these challenges. For future study, we will consider Ensembling all variants of EfficientNets and compare with other state-of-the-art models for detecting foreign objects on CXR images that could affect performance or be misclassified as TB. Although the proposed methodology is specific to TB detection, it could be extended to detect other pulmonary abnormalities, given the right set of datasets.

## Figures and Tables

**Algorithm 1:**
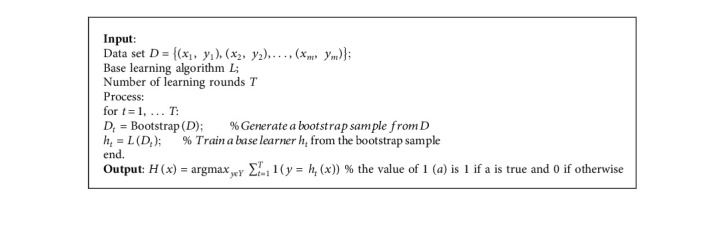
Algorithm 1 Bagging Ensemble.

**Table 1:** Data augmentation types and probability values.

Augmentation type	Probability value
Rotation_right_left	0.5
Zoom range	0.3
Vertical flip	0.4
Horizontal flip	0.4
Height_shift_range	0.2
Width	0.2

**Table 2:** CLAHE parameter values.

Parameter	Value
clipLimit	2.0
tileGridSize	8 × 8

**Table 3:** Experimental results.

EfficientNet model	Montgomery				Shenzhen			
	Accuracy (%)	Sensitivity (%)	Specificity (%)	AUC	Accuracy (%)	Sensitivity (%)	Specificity (%)	AUC
B0	80.52	82.10	81.48	0.77	84.90	87.08	85.36	0.82
B1	83.46	83.78	83.77	0.80	86.62	89.12	90.81	0.86
B2	86.35	90.16	88.25	0.84	91.24	90.83	90.31	0.90
B3	90.40	93.01	92.00	0.90	92.83	93.64	89.53	0.92
B4	92.33	95.41	93.61	0.92	94.35	95.69	94.15	0.93
Ensemble	95.82	98.13	95.78	0.94	97.44	99.18	96.21	0.96

**Table 4:** Parameter values and input sizes of each EfficientNet.

Model	Parameter (million) (M)	Input size
B0	5.3	224 × 224
B1	7.8	240 × 240
B2	9.2	260 × 260
B3	12	300 × 300
B4	19	380 × 380

**Table 5:** Proposed method compared with the related Ensemble study.

Author	Result (%)
Lopes and Valiati [21]	84.6
Rajaraman and Antani [23]	94.1
Hernández et al. [17]	86.4
Hijazi et al. [24]	89.7
Dasanayaka and Dissanayake [11]	97.1
Pasa et al. [10]	86.2
Sahlol et al. [29]	94.1
Proposed method	97.4

## Data Availability

Data used in this research are publicly available (Shenzhen and Montgomery TB-specific CXR datasets). The developed model can be shared upon request.
